# Hospital Progress and Finance

**Published:** 1923-04

**Authors:** 


### HOSPITAL PROGRESS AND FINANCE.

### THE AFFAIRS OF THE SUSSEX COUNTY HOSPITAL.

JUST after our March number went to press, the report of the committee of investigation into the finance and affairs of the Royal Sussex County Hospital was at last issued. The committee, which had been appointed last October, expresses regret that the board did not consult an expert early in 1922, when it became clear that the expenditure was largely in excess of the hospital's income. Statistics are given to show that, while the cost per head in a group of hospitals fell £3, that of a bed in the Sussex County Hospital increased £15. On the domestic side of the accounts, the expenditure was 32 per cent. above the cost in kindred institutions, and the surgery and dispensary showed an advance of 38 per cent. The committee observed a great want of control in the dispensary, and strongly recommended that the supervising powers of the superintendent should be strengthened. In regard to appeals the committee advises joint action with other charities in the district, and suggests that a central committee should be formed, on which each institution should be represented. The collection of money would be regulated by district sub-committees. So much was hoped from the Sussex Provident scheme that the subsequent financial difficulties of the County Hospital were felt to be the more surprising. It appears that there has been a want of control over expenditure, but since the hospital was eager to set its house in order, and started the investigation by experts, it will be charitably judged if the recommendations are promptly acted upon and early improvement is recorded. A summary of the report appears on another page.

---

### THE LONDON HOSPITAL'S QUINQUENNIAL YEAR.

IN an interesting speech at the Court of Governors Lord Knutsford, Chairman of the London Hospital, referred to Queen Alexandra, the hospital's President. She had started the Finsen Light department, where it was found that the light had a beneficial effect on people suffering from other complaints who sat round it. It was therefore hoped to extend its use. A ward had been converted into an improved X-ray department about to be opened, thanks to a gift from the East End Tradesmen's Association. This is the year when the quinquennial appeal falls due, and the hospital, said the Chairman, would practically have to run for five years on the proceeds. Payments from in-patients had produced £21,746 during the year. Sixty per cent. of the in-patients paid the charge of a guinea a week. The remainder paid nothing, or a fraction only; the guinea represented one-fifth of the cost of each patient. The present year is not a very favourable one for a big effort like the quinquennial appeal, and every sympathy will be felt with its organisers. After so much has been attempted in London, and with simultaneous efforts proceeding, the appeal will be a severe test of the hospital's now famous enterprise in income-raising.

---

### THE APPROVED SOCIETIES' SURPLUS.

IN some letters to the "Manchester Guardian," Mr. Stafford Galland has been discussing the surplus of the Approved Societies in regard to hospitals. The valuation of the societies' funds, he says, is calculated on the basis of interest at 3 per cent., any interest on investments above this being a profit; the societies, who are exempt from income tax, have invested in Government securities, so that "the high rate of interest is coming out of the pockets of the tax-payer." He suggests, therefore, that, as the funds of Health Insurance are not contributed by members only, but largely by employers and the State, the high rate of interest earned in the investment of the funds contributed by employers should be subject to income tax. The Health Insurance Fund would still receive, he argues, not less than the actuarial 3 per cent.; and the amount raised by the tax should be allotted to the hospitals on the basis of the Onslow Report. The suggestion that this would satisfy everyone leaves out of account the Approved Societies, but, as they can well take care of themselves, the opinion of the hospitals and the Government would be of interest.

---

### FLEET STREET AND BART'S.

IT has been decided to hold a Fleet Street Week in the autumn for the benefit of St. Bartholomew's Hospital. The arrangements are in the hands of a committee representing the various Press associations and clubs. Last year a Fleet Street committee raised £7,000 for the hospital, but on this occasion a larger sum is aimed at. We hope it may prove possible to create a permanent body that will collect an annual sum, since the associations concerned should be as susceptible of organisation as any other business concern.

---

### SICK PERSONS IN POOR LAW HOSPITALS.

THE Barnet Board of Guardians, who are responsible for the Wellhouse Hospital, are endeavouring to secure the removal of the disqualifications that still attach to patients in Poor Law hospitals, but which would not exist if these hospitals were under the control of any other public authority. In correspondence with the Ministry of Health, the Guardians urge that the removal of these disqualifications should not wait for the long-expected scheme for the reform of the Poor Law. The familiar grievance —which has become acute since the Poor Law hospitals have been officially encouraged to come to the assistance of the voluntary hospitals because of the latter's long waiting lists and scarcity of beds—affects two classes of people. One is the sick poor, who become "paupers" if they receive treatment in Poor Law hospitals, even if this is paid for, and pensioners who forfeit their pensions on admission. The second is the class of Guardians or Councillors who become disqualified under penalty for the performance of their duties if they or their families receive treatment. The Barnet Guardians and all sensible people condemn this anomaly, but since old practices die hard it is well to be instant in season and out of season that the inevitable change may be unflaggingly advocated.

### ACTORS AND THE HOSPITAL SAVING ASSOCIATION.

At a recent meeting of the Council of the Actors' Association, Mr. R. J. Coles, of King's College Hospital, explained the objects of the Hospital Saving Association. The object of the scheme was to reopen closed wards. All weekly contributions went to a central fund, which made payments to any hospital, so that no contributor would be confined to one. In reply to questions, Mr. Coles said that the same procedure applied to hospitals outside London; the central fund would make a reasonable contribution toward the cost of a contributor's treatment; and that no time limit had been fixed before he became eligible for help, at least where he was a member of a collective body. Mr. Maurice Hoffman recommended the scheme to actors and actresses, and a resolution was carried recommending the Council to appoint a committee to investigate the possibilities of the Actors' Association joining the Hospital Saving Scheme.

### A SUCCESSFUL INSURANCE SCHEME.

The so-called insurance scheme connected with the Royal Hampshire County Hospital at Winchester, with which Mr. L. B. Keyser, the Treasurer, has been so actively associated, produced last year a sum of £6,109, or nearly twice the total raised in 1921. Those who do not subscribe to the hospital at present, but approve of the contributory scheme, are invited to become honorary members of it. A sum of £3,105 has been received from patients who are not contributors; and, of course, it is clear that it would be to the general advantage if this item did not recur and the number of contributors increased proportionately. The number of persons insured is 19,000, and the hospital was recently able to reduce the family rate of contribution from 6d. to 4d. per week, and to include children in the 2d. a week paid by a widower or widow. Additional benefits granted were free maintenance in any cottage hospital, and, when ordered by the hospital staff, free convalescent treatment.

### HOSPITAL SHIPS IN AFRICA.

We have grown accustomed to think of hospital ships as of value only in time of war, but a recent accident to the daughter of the retiring Governor-General of the Belgian Congo reminds us how useful they are in those remote places where the rivers are the principal highways. A motor accident happened to the Governor-General and his daughter about 1,400 miles from the capital, and the latter was severely injured. There were, says a "Times" correspondent, no medical conveniences, no nurses and very little proper food. As the travellers were near the Sudan frontier, the authorities were somehow informed, and despatched a special car fitted with a bed which carried the patient three hundred miles to Rejaf, where the party was met by the Lady Baker Hospital Ship, which carried them 1,500 miles. Without the assistance of the Sudan authorities, the Governor-General declares that he could never have brought his daughter home alive. His wife has lately raised a fund in Belgium to place a similar hospital ship upon the Congo, and it is remarkable that the Governor-General should be the first person to prove to the Belgians what humane and valuable work such a ship can accomplish.

### A HOSPITAL SYSTEM FOR SOMERSET.

A very interesting and suggestive report has been issued by the Local Committee for Somerset (excluding Bristol which has its own Local Committee). It divides the county into three clearly defined hospital areas, and urges that if the north-west of the county is to have a co-ordinated hospital service, the initiative must come from Bristol—"the area badly needs organisation." A Bath Committee is also strongly advocated. The report contains a summary of the defects in the existing hospital system. The most important of these are inadequate hospital accommodation; excessive concentration of patients in Bristol and Bath; lack of co-ordination between the general hospitals of Bristol and Bath, and between these and the cottage hospitals; lack of accommodation for patients of moderate means; the need of maternity and convalescent homes, of an ambulance service, and a general failure to realise that a modern hospital service must provide not only for the very poor but for all willing to pay, in their degrees, for it. This is the most comprehensive and cogent report by any Local Committee that we have seen.

### GLOUCESTER WORKMEN'S FUND.

Very remarkable progress has been made with the Workpeople's Hospital Fund in Gloucester. In 1921 it raised £2,500, and last year £4,900 or nearly double. Mr. Hogg, the honorary secretary, records that the list of contributing bodies contains the names of 332 groups; the miners in the Forest of Dean are joining the scheme in larger numbers, and when we remember that Gloucester is a western cathedral city, and therefore supposed to be the reverse of an industrial town, the result is one that would have been deemed impossible a few years ago, when funds of this kind were almost exclusively confined to manufacturing centres. We long had to combat the idea that they could not be transplanted to the south and west of England.

### A NEW HOSPITAL FOR CARDIFF.

It is stated that the Cardiff Board of Guardians proposes to build a new infirmary at a cost of £250,000. The site, at Llandough, has been bought for £1,750 from the Marquess of Bute; and the plans of the building are understood to be designed on an original scheme.

